# 六溴环十二烷的样品前处理和检测方法研究进展

**DOI:** 10.3724/SP.J.1123.2022.03030

**Published:** 2022-10-08

**Authors:** Jiawen CHENG, Jiping MA, Shuang LI, Yong TIAN

**Affiliations:** 青岛理工大学环境与市政工程学院, 山东 青岛 266525; School of Environmental and Municipal Engineering, Qingdao University of Technology, Qingdao 266525, China

**Keywords:** 样品前处理, 检测方法, 六溴环十二烷, 溴代阻燃剂, 综述, sample pretreatment, detection methods, hexabromocyclododecanes (HBCDs), brominated flame retardants, review

## Abstract

作为一种常见的溴代阻燃剂,六溴环十二烷(HBCDs)因具备持久性、长距离迁移性、生物蓄积性和高毒性,于2013年被列入《斯德哥尔摩公约》。因此,环境样品中HBCDs污染水平的准确分析和严格控制对完善环境监管长效机制至关重要。然而,实际样品中HBCDs的定性定量分析正面临着基质复杂、目标物含量低等问题。尤其,HBCDs在高温环境及特定有机溶剂中易降解,会产生异构体,提高了分析难度。该综述简述了HBCDs的理化性质、毒性危害和标准限制,重点围绕不同基质中HBCDs的样品前处理和仪器检测两方面进行了总结。论文内容引用2000~2022年的70余篇源于科学引文索引(SCI)与中文核心期刊中的相关论文。总结归纳了固体和液体样品中HBCDs分析的前处理技术,包括索式提取、超声辅助萃取、加速溶剂萃取、超临界流体萃取、液液萃取、分散液液微萃取、固相萃取、分散固相萃取和固相微萃取等,介绍了气相色谱、液相色谱和色谱-质谱联用技术等仪器检测方法在HBCDs分析中的应用。通过综述近期相关研究,侧面表明HBCDs的分析方法研究发展迅速,但也面临一些挑战,如样品前处理步骤繁琐、耗时长、样品量和有机溶剂用量大等问题。最后,对新型样品前处理技术在HBCDs分析中的应用进行了展望。

## Abstract (en)

Hexabromocyclododecanes (HBCDs) are a group of brominated flame retardants that are extensively employed in the industrial production of plastics, furniture, and construction materials. Due to their regular use and massive emissions, HBCDs have been distributed in the environment (air, water, soil, and sediments). Due to their high toxicity, persistent and long-distance transport, and bioaccumulation, HBCDs were listed in the Stockholm Convention in 2013. Thus, the accurate analysis and strict control of the pollution levels of HBCDs in environmental samples are critical to the government’s long-term environmental supervision mechanism. However, the concentration levels of HBCDs in real samples are low, combined with complex matrices, which seriously limits the determination of HBCDs. The target isomers are particularly transformed or degraded, which makes analysis challenging due to the high temperatures and specific organic solvents. The physicochemical features, toxic and environmental dangers, usage, and standard limitations of HBCDs are briefly discussed herein. Sample pretreatment and instrument detection of HBCDs in various matrices are summarized (i. e., soil, sediment, food, electronics, atmosphere, animals, and water). More than 70 research papers (2000-2022) from the Science Citation Index (SCI) and Chinese core publications are cited herein. First, the entire process of extraction, purification, separation, and enrichment of HBCDs is compared, including soxhlet extraction (SE), ultrasonic-assisted extraction (UAE), accelerated solvent extraction(ASE), supercritical fluid extraction (SFE), solid phase extraction (SPE), dispersed solid phase extraction (DSPE), liquid-liquid extraction (LLE), dispersive liquid-liquid microextraction (DLLME), and solid phase microextraction (SPME). In the literature, UAE is the most commonly employed process, accounting for a quarter of all HBCDs sample pretreatments. Additionally, SPE sample pretreatment technology can completely separate the targets and impurities to reduce the effect of matrix; and enrich the targets to improve sensitivity of method. By using SPE technology, the enrichment factor can be increased due to the massive sample volume, thus enabling detect HBCDs concentrations (nanogram per liter level) in water. Second, the advantages and disadvantages of instrument approaches are examined and discussed, including gas chromatography (GC), gas chromatography-mass spectrometry (GC-MS), liquid chromatography (LC), and liquid chromatography-tandem mass spectrometry (LC-MS/MS), which offer a reference for meeting the requirements in the determination of HBCDs in real samples, i. e., the total HBCDs can be detected by GC or GC-MS and three HBCD isomers (*α*-HBCD, *β*-HBCD, *γ*-HBCD) can be determined by LC or LC-MS. By using the triple quadrupole mass spectrometer, the sensitivity of the method can be improved. Till date, LC-MS/MS has been chosen as an instrument for the determination of HBCDs in various matrices (i. e., seawater, marine sediment, marine organisms, toys, and electronic products) in China. However, there are several challenges. The sample pretreatment is tedious; large sample volumes and organic solvent utilization. These challenges point to the development trends of analytical approaches for HBCDs. The development of green, automated, low-cost, fast, and efficient sample pretreatment approaches for new adsorption materials is the main development direction in the analysis of HBCDs in the future.

六溴环十二烷(hexabromocyclododecanes, HBCDs)是一种通过环十二烷三烯溴化反应而成的溴代阻燃剂^[1]^。理论上,共存在16种HBCDs立体异构体,其中3种为主要的商品化HBCDs,分别为*α*-HBCD(10%~13%)、*β*-HBCD(1%~12%)和*γ*-HBCD(75%~89%)^[2]^,它们的结构式及理化性质如表1所示。据市场调研,2001年HBCDs的全球产量为16700 t^[4]^,到2011年其全球产量增至28000 t。作为典型溴代阻燃剂的代表之一,HBCDs被广泛应用于电子产品、家具和建筑材料中^[5]^。但是,毒理学研究表明,HBCDs具备内分泌干扰作用、发育神经毒性和甲状腺毒性,会诱导基因重组引发癌症等疾病^[6⇓-8]^。此外,HBCDs具有强疏水性(log *K*_ow_=5.38~5.80)、生物蓄积性^[9]^、长距离迁移性与持久性^[10]^。2008年,欧盟《关于化学品的注册、评估、授权、限制》(REACH)法规规定进入欧盟市场的物品中HBCDs的含量不得超过0.1%。美国环境保护署(EPA)将HBCDs加入至《有毒物质控制法》(TCSA)中,规定在生产或进口HBCDs前至少90 d向EPA提交申请。2013年,HBCDs被列入《斯德哥尔摩公约》的持久性有机污染物清单中,同年欧盟对EC 850/2004进行修订,禁止含有超过100 mg/kg HBCDs的物质进入欧盟市场。2016年,加拿大将HBCDs添加至《加拿大环境保护法中》,在建筑或工程用途中禁止生产、使用、销售、供应和进口含有HBCDs的发泡聚苯乙烯和挤塑聚苯乙烯。2018年,我国将其列为优先限用物质^[11]^。

**表1:** HBCDs的结构式及理化性质^[3]^

Chemical	Structure	Water	log	Boiling
		solubility/(μg/L)	K_ow_	point/℃
α-HBCD	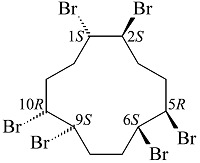	48.8	5.38	190
β-HBCD	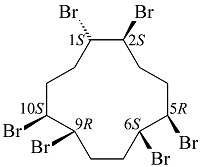	14.7	5.47	190
γ-HBCD	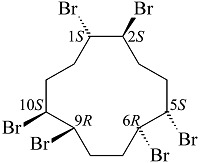	2.1	5.8	190

尽管世界各地已对HBCDs做出严格限制,但在建筑行业其具有特定的豁免权,可作为阻燃剂用于建筑物中^[11]^。所以,塑料、建材和建筑垃圾等将成为人体暴露HBCDs的关键来源。此外,由于特殊的物理及化学性质,HBCDs可能迁移至水体中,随之进入全球径流^[12]^,部分被海洋生物吸收从而随食物链蓄积最终危害人体健康^[13]^。目前,HBCDs已经在发泡聚苯乙烯材料^[14,15]^、食品^[16]^、土壤^[17]^、环境大气^[18]^、母乳^[19]^、血浆^[20,21]^、尿液^[22]^、生物^[23,24]^、沉积物^[25,26]^、粉尘^[27]^、环境水体^[28]^中被频繁检出。鉴于HBCDs无处不在以及3种主要异构体之间的相互转化^[10]^,对其进行准确定性定量分析尤为重要。但实际样品中HBCDs的含量较低(ng/L~μg/L或ng/kg~μg/kg),且复杂的基质干扰加大其分析测定的难度,因此,需对样品进行萃取、净化、提纯和浓缩,并结合高灵敏度检测仪器,建立高效的分析方法。目前,HBCDs的前处理方法有索式提取、超声辅助萃取、加速溶剂萃取、固相萃取、液液萃取等,其中索式提取、超声辅助萃取和加速溶剂萃取等多用于固体样品中HBCDs的萃取,而液液萃取等主要起到了液体样品中HBCDs分离富集的作用;HBCDs的仪器检测方法主要是气相色谱、液相色谱、气/液相色谱-质谱联用。

近几年关于HBCDs相关综述多侧重报道生物和大气中HBCDs的分析方法^[29]^及其污染水平和来源^[5,30⇓-32]^。在此基础上,本文侧重总结和讨论土壤、沉积物、食品、电子产品、大气、动物、水体等不同基质中HBCDs的样品前处理方法和仪器检测方法的研究进展,并对该领域未来发展进行了展望。表2列出了文献报道的不同基质中HBCDs的样品前处理方法和仪器检测方法及其检出限、精密度和加标回收率。

**表2:** HBCDs的分析方法与性能

Matrices	Analyte	Pretreatment	Instrument	LOD	RSD/%		Recovery/		Ref.
		method					%		
Surface sediment	ΣHBCDs	UAE	GC-MS	-		<20	60	-120	[25]
Polymers of electrical and electronic	α,β,γ-HBCD	UAE	LC-MS	1 mg/kg	4.5	-4.7	88.3	-104.5	[33]
products									
Seabird eggs	α,β,γ-HBCD	UAE	LC-MS/MS	-		-	84	-169	[34]
Seabird eggs	ΣHBCDs	UAE	GC-MS	-		-	84	-169	[34]
Plastic toys	α,β,γ-HBCD	UAE	LC-MS/MS	0.010-0.012 mg/kg	3.8	-9.9	98.1	-96.2	[35]
Sea water	α,β,γ-HBCD	LLE+SPE	LC-MS/MS	2 ng/L		<20	70	-120	[36]
Water	α,β,γ-HBCD	DLLME	LC-MS/MS	84.6-156.4 ng/L	5.3	-9.7	71	-102	[37]
Water	α,β,γ-HBCD	DLLME	LC-MS/MS	100 ng/L	2.04	-8.43	77.2	-99.3	[38]
Water	α,β,γ-HBCD	DLLME	LC-MS/MS	0.12-0.22 μg/L	4.1	-6.7	88	-114	[39]
Marine organisms	α,β,γ-HBCD	SPE	LC-MS/MS	0.06 μg/kg	3.9	-8.8	85.6	-97.3	[40]
Dust	α,β,γ-HBCD	SPE	LC-MS	2-10 pg		-	60	-120	[41]
Water	α,β,γ-HBCD	SPE	LC-MS/MS	0.5 ng/L	4.3	-9.3	78.8	-85.1	[42]
Marine organisms	α,β,γ-HBCD	UAE+SPE	LC-MS/MS	0.2 μg/kg		<20	70	-120	[43]
Marine sediment	α,β,γ-HBCD	UAE+SPE	LC-MS/MS	0.2 μg/kg		<20	70	-120	[44]
Fish	α,β,γ-HBCD	DSPE	LC-MS/MS	0.030-0.075 μg/kg	2	-12	86	-115	[45]
Water	α,β,γ-HBCD	SPME	LC-MS/MS	0.01-0.04 ng/mL	6.45	-10.7	88	-108	[46]
Sewage sludge	α,β,γ-HBCD	UAE+DSPE	LC-MS/MS	0.2-0.3 ng/g	1.86	-8.79	79.6	-112.5	[47]
Sediment	α,β,γ-HBCD	SE	LC-MS/MS	-		-		-	[28]
Sediment	ΣHBCDs	Derivatization	GC-MS	-		-		65	[48]
Fish	α,β,γ-HBCD	ASE	LC-MS/MS	-		-	61	-106	[49]
Fish	α,β,γ-HBCD	ASE	LC-MS/MS	0.03 μg/kg	7	-19	70	-110	[50]
Suspended particulate matter/	α,β,γ-HBCD	ASE	LC-MS/MS	0.3 μg/kg	3	-4	70	-110	[50]
sediment									
Bird	α,β,γ-HBCD	ASE	LC-MS/MS	-		-	83	-105	[51]
Soil	ΣHBCDs	SFE	GC-MS	0.4 mg/kg	0.52	-1.5	93.2	-99.6	[52]
Electrical and electronic products	ΣHBCDs	UAE	GC	3 mg/kg	2.1	-5.6	78.8	-106	[53]
ABS	ΣHBCDs	UAE+SPE	GC-MS	10 mg/kg	4.3	-8.4	99	-102	[54]
Water	ΣHBCDs	SPE	GC-MS	1 mg/L	3.6	-5.7	91.3	-108	[55]
Plastic waste	ΣHBCDs	UAE+QuEChERS	GC-MS	-		-	96	-118	[56]
Polystyrene	α,β,γ-HBCD	-	LC	-		0.7	90	-95	[57]
Ambient air	α,β,γ-HBCD	SE+GPC	LC-MS	0.4-0.5 μg/L	3.6	-8.9	74.8	-95.8	[18]
Soil	α,β,γ-HBCD	ASE+SPE	LC-MS/MS	1.8-2.8 ng/kg	5.8	-11.2	73.8	-106.9	[17]
Textiles	α,β,γ-HBCD	SE	LC-MS/MS	2.9-5.9 μg/kg	1.84	-4.81	85	-103	[58]
Toys	α,β,γ-HBCD	SE/UAE	LC-MS/MS	5 mg/kg	4.7	-22.1		-	[59]
Electrical and electronic products	α,β,γ-HBCD	SE/UAE/ASE	LC-MS/MS	20-30 mg/kg		-	70	-120	[60]
Porphyra, kelp and sargassum	α,β,γ-HBCD	SE+SPE	LC-MS/MS	10 μg/kg			80.1	-104.1	[61]
Soil and Sediment	α,β,γ-HBCD	SE/ASE+GPC	LC-MS/MS	9.95-12.5 ng/kg	4.1	-11	78.5	-111	[62]
Soil	α,β,γ-HBCD	ASE+GPC	LC-MS/MS	0.1-1.2 pg/g	1.8	-3.9	81.8	-113	[63]
Aquatic products	α,β,γ-HBCD	QuEChERS	LC-MS/MS	0.04-0.16 μg/kg	0.42	-23	80.5	-113	[23]
Technical HBCDs mixture	α,β,γ,δ,ε,ζ,	-	LC-MS/MS	-		-		-	[64]
	η,θ,ι,κ-HBCD								

*α*,*β*,*γ*-HBCD and *α*,*β*,*γ*,*δ*,*ε*,*ζ*, *η*,*θ*,*ι*,*κ*-HBCD means individual isomers; UAE: ultrasonic-assisted extraction; LLE: liquid-liquid microextraction; DLLME: dispersive liquid-liquid microextraction; SPE: solid phase extraction; DSPE: dispersed solid phase extraction; SE: soxhlet extraction; GPC: gel permeation chromatography; ASE: accelerated solvent extraction; SFE: supercritical fluid extraction; ABS: acrylonitrile butadiene styrene copolymers; -: not mentioned.

## 1 固体样品前处理方法

### 1.1 萃取

#### 1.1.1 索式提取

索式提取作为传统的前处理方法,在多种基质中应用广泛,其萃取效率的关键在于萃取溶剂中目标物的溶解度以及萃取溶液与基质的接触程度^[65]^。Zhong等^[65]^总结了2002~2016年间,HBCDs索式提取使用的萃取溶剂(如图1所示)。结果表明,丙酮/正己烷混合溶液作为萃取溶液的使用频率最高。但文中指出HBCDs在丙酮中不稳定,能够与丙酮反应形成其他溴代产物,因此他们重新评估了丙酮作为HBCDs索式提取溶液的可行性。研究考察丙酮、丙酮/正己烷(3∶1, v/v)和丙酮/正己烷(1∶1, v/v)作为溶剂时的萃取效率,分别为78%、70%和94%,当萃取时间从24 h增加至72 h时,回收率分别降至55%、5%和13%,回收率的下降可能归因于长时间的萃取过程使HBCDs大量损失,并且在丙酮/正己烷混合溶液中检出了多溴联苯醚,而纯丙酮萃取液中未检出。结果表明,正己烷的加入会导致HBCDs降解中间体的生成,这一结果可能会误导环境研究者们对于HBCDs环境归趋的研究^[65]^。因此,该项研究中建议使用甲苯、二氯甲烷、甲醇、乙酸乙酯、甲基叔丁基醚、环己烷、异丙醇和正己烷作为HBCDs索式提取的萃取溶剂。

**图1:**
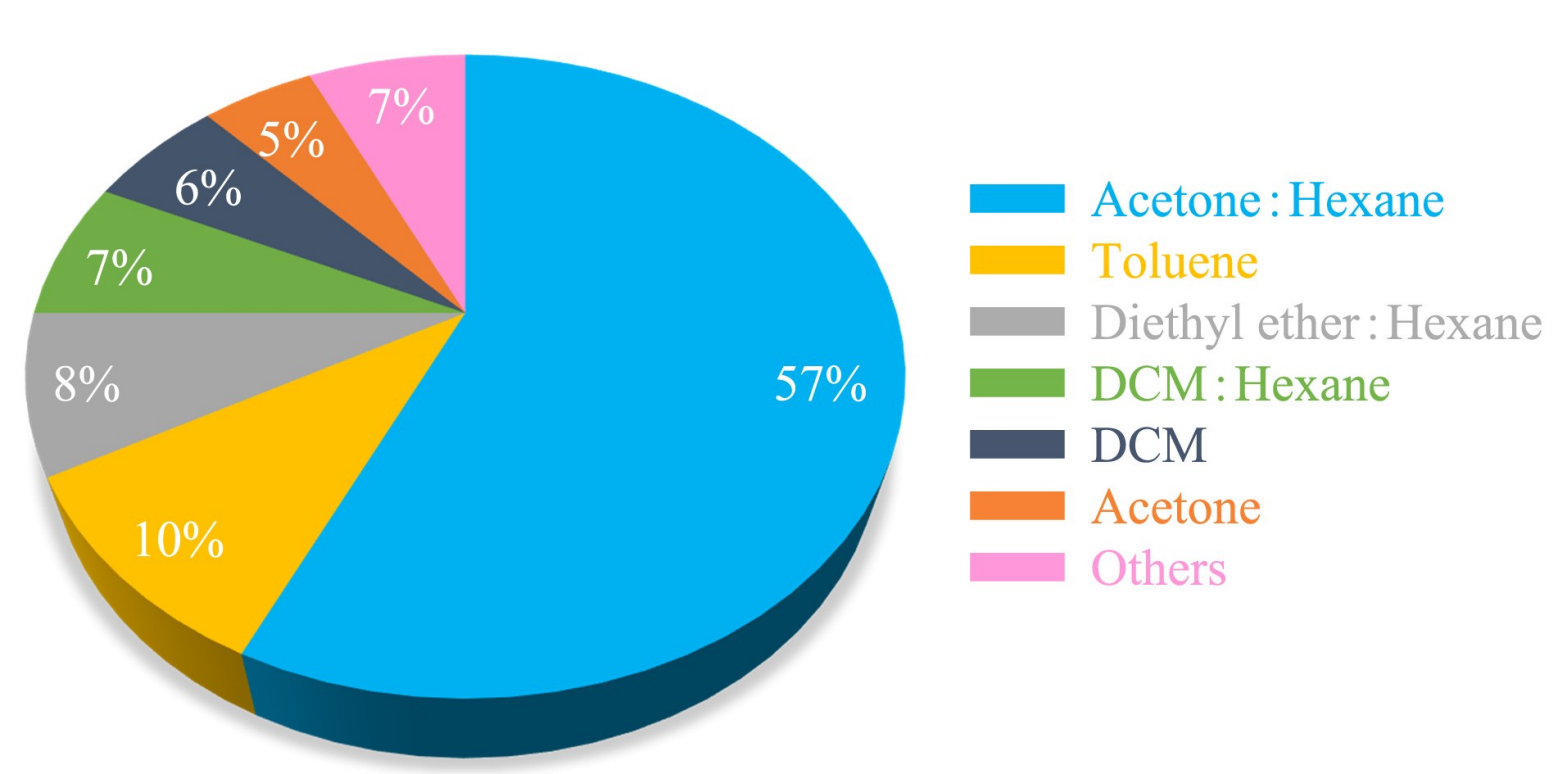
索式提取作为HBCDs前处理方法所用萃取溶剂总结 (来源:Web of Science, 2002~2016)^[65]^

#### 1.1.2 超声辅助萃取

超声辅助萃取(UAE)传质快,可以在短时间内获得萃取液。萃取溶剂和萃取时间是提高萃取效率的主要因素。陈琼等^[33]^在对聚丙烯样品中的HBCDs提取时,发现甲苯表现出较高的萃取效率,并且萃取可以在5 min内完成。不同于索式提取,短时间的萃取会减少丙酮引起的HBCDs降解,Hoang等^[25]^选择丙酮/正己烷(1∶1, v/v)作为萃取溶剂,仅耗时10 min即可完成对表层沉积物中HBCDs的萃取,回收率在60%~120%之间。通常,处理小体积样品时,超声辅助萃取简单、方便、快速,也可以采用连续式或循环式超声辅助萃取处理大批量样品。

#### 1.1.3 加速溶剂萃取

加速溶剂萃取(ASE)基于高温高压条件,使萃取溶剂充分接触样品,从而提高萃取效率^[66]^。Letcher等^[51]^在0.07 MPa、100 ℃下使用二氯甲烷/正己烷(1∶1, v/v)对红隼肝脏和脂肪组织中的HBCDs进行加速溶剂萃取,在0.5 h内可以完成萃取。相同的高温高压条件,Rüdel等^[50]^使用二氯甲烷萃取悬浮颗粒物中的HBCDs,回收率可以达到70%~110%。加速溶剂萃取对固体样品有较高的提取效率,并且方法重复性好,准确度高,全程自动化操作。与索氏提取相比,该方法可以大大缩短固体样品的萃取时间,且可实现与索氏提取相当的回收率。

#### 1.1.4 超临界流体萃取

超临界流体萃取(SFE)利用超临界流体在特定的温度和压力下将目标物从基质中提取出来,二氧化碳(CO_2_)作为超临界流体的首选,具有无毒、不易燃和环境友好的特点^[52]^。在萃取过程中,纯CO_2_会优先萃取非极性和亲脂性物质,但HBCDs为弱极性物质,萃取效率可能会受到影响,因此,Wang等^[52]^研究了温度、压力、表面活性剂和有机改性剂对萃取效率的影响。结果表明,当萃取条件为50 ℃、25 MPa、添加2%表面活性剂(Triton X-14)和20%乙醇时,萃取效率达到最高。这可能是因为高温高压可以增加HBCDs在CO_2_中的溶解度和传质速率,但过高的温度和压力促使CO_2_密度降低,黏度提高,严重影响萃取效率^[52]^。2%的表面活性剂能够在CO_2_和HBCDs中起到架桥作用,增加了两种物质的接触^[52]^。此外,20%乙醇也可增加HBCDs的溶解度,从而提高萃取效率。将优化的分析方法应用于土壤中HBCDs的测定,加标回收率达到98.3%~99.3%,并在6份土壤样品中均有HBCDs检出。

### 1.2 净化

固体样品经萃取后,还需要进一步的净化才能将提取液中的目标物与杂质分离,降低基质效应,在仪器检测时提高灵敏度^[67]^。

#### 1.2.1 固相萃取

固相萃取(SPE)的净化主要是利用固相萃取柱将固体或液体样品提取液中的杂质进行去除,从而获得较为纯净的目标物。常用于净化的固相萃取柱有硅胶柱、硅酸镁柱和氨基柱等^[68]^。目前,HBCDs的相关行业标准(HY/T 259-2018、HY/T 260-2018)采用SPE作为前处理方法^[63,64]^,标准中使用硅胶柱对海洋生物和海洋沉积物的提取液进行净化处理,3种HBCDs的定量限均为0.2 μg/kg。严忠雍等^[40]^采用硅胶固相萃取小柱对海洋生物中HBCDs的提取液进行净化,并考察了适用于硅胶柱的洗脱溶剂(环己烷、二氯甲烷、乙腈、甲醇)对HBCDs萃取效率的影响。结果显示,环己烷极性较弱,对于强极性填料的硅胶柱来说,无法有效地从柱中分离出HBCDs。而甲醇极性较强,但洗脱能力过强导致洗脱液杂质过多,对仪器检测造成干扰。为了提高分析效率,选择了易挥发的二氯甲烷作为洗脱溶液。

#### 1.2.2 分散固相萃取

与固相萃取柱不同的是,分散固相萃取(DSPE)将吸附剂分散于样品中,可增加与目标物的充分吸附。Lankova等^[45]^采用分散固相萃取法将100 mg C18吸附剂和20 mg伯仲胺吸附剂分散于基质溶液中,用于萃取鱼肉中的HBCDs,回收率可达98%。Anastassiades等^[69]^提出一种基于分散固相萃取的新型样品前处理方法(QuEChERS),该法具备快速(quick)、容易(easy)、经济(cheap)、有效(effective)、稳定(rugged)和安全(safe)的特点。水产品样品中含有大量的脂肪、有机酸和色素等干扰物,需要选择适当的吸附剂以去除干扰物,提高分析准确性。C18适合去除脂肪等弱极性干扰物;PSA为*N*-丙基乙二胺,适于去除极性物质、有机酸、色素和金属离子;GCB为石墨化炭黑,适于去除色素。于紫玲等^[23]^考察了C18、PSA和GCB作为吸附剂对鱼类水产品中四溴双酚A和HBCDs的萃取效率。研究发现,虽然GCB能够去除色素,但其对含有苯环官能团的目标物(四溴双酚A)具有较强的吸附能力,因此GCB不作考虑;而PSA多用于有机酸和色素含量高的植物农残品的处理,但鱼类水产品的脂肪含量高,脂肪的干扰大于有机酸和色素的干扰,所以选用C18较为适合。结果表明,使用C18作为吸附剂时,该法对HBCDs的回收率最高,可达80%~100%,因此研究最终选择了将50 mg无水硫酸镁和50 mg C18置于萃取液中进行涡旋离心,取上清液进行检测。

## 2 液体样品前处理方法

### 2.1 液液萃取

目前我国海洋行业标准(HY/T 261-2018)采用液液萃取(LLE)对海水中的HBCDs进行萃取^[36]^,该法使用40 mL正己烷分两次对500 mL的海水样品进行液液萃取,浓缩提取液后加入5 mL正己烷复溶,将复溶后的溶液通过硅胶柱进行净化,最后洗脱液经氮吹后使用1 mL甲醇复溶,富集倍数为500,最终该方法的定量限为0.002 ng/mL。

### 2.2 分散液液微萃取

分散液液微萃取(DLLME)具备操作简单、快速、回收率和富集倍率高、样品和溶剂用量少等特点,是一种环境友好型样品前处理技术^[70]^。温控离子液体分散液液微萃取将“绿色”溶剂离子液体作为萃取剂,不会对环境造成污染且能溶解无机物、极性和非极性有机物^[37]^。根据温度的变化使离子液体完全分散在水相中,再通过冷却和离心将离子液体析出,使其浓缩成一滴^[39]^。该法的重点是离子液体种类和溶解温度的选择。齐鲁工业大学赵汝松课题组^[39]^根据“相似相溶”原理,考虑到HBCDs是弱极性化合物,鉴于离子液体的极性,选择[C_8_MIN][PF_6_]作为离子液体,对雪水、雨水、河水和湖水等环境水体中的HBCDs进行萃取。除了离子液体种类以外,适当的溶解温度可使其完全分散在水相中,该课题组在另一项工作^[38]^中发现在75 ℃时,该法对HBCDs的萃取效率达到最大,而随着温度上升萃取效率逐渐下降。温控离子液体分散液液微萃取作为液液萃取的替代方法,具有富集倍数高(500~1000倍)、检出限低、样品用量少(5 mL)、萃取时间短等优点。

### 2.3 固相萃取

对于液体样品而言,SPE主要是富集浓缩的作用,固相萃取具有回收率高、萃取时间短、富集倍数高、有机溶剂用量少、易于自动化操作等优势^[71,72]^。固相萃取柱的填料种类繁多,Han等^[42]^考察了MAX、p-Pak C18、Bond Elut C18和HLB 4种小柱对环境水样中HBCDs的萃取效果,其中MAX为混合型阴离子交换柱,HLB小柱的吸附剂是亲水亲脂的乙二烯苯/*N*-乙烯基吡咯烷酮共聚物。而HBCDs在自然条件下为分子形态,MAX对其萃取效率较低;HLB的吸附容量是C18的3.3倍^[73]^,使得在相同的样品体积下,HLB的萃取回收率较高,因此选择了HLB对环境水样中的HBCDs进行分离富集,检出限可达0.5 ng/L。SPE能够对大体积水样中的目标物进行萃取,与其他前处理方法相比,检出限可降低1~2个数量级(如表2所示)。

### 2.4 固相微萃取

固相微萃取(SPME)集采样、浓缩、直接进样于一体^[74]^,缩短了前处理时间,从而提高分析效率。Yu等^[46]^建立了直接浸没固相微萃取法用于水中HBCDs的萃取(如图2所示)。将商品化的固相微萃取纤维(涂层:聚二甲基硅氧烷/二乙烯基苯)浸没于固相微萃取装置中,利用六通阀连接固相微萃取装置、流动相(洗脱溶剂)、检测仪器与废液。通过调节六通阀完成对水中HBCDs的萃取和富集,该法LOD为0.01~0.04 ng/mL, RSD为7.47%~10.70%。该法将前处理设备与检测仪器连接起来,实现了HBCDs的在线分析。

**图2:**
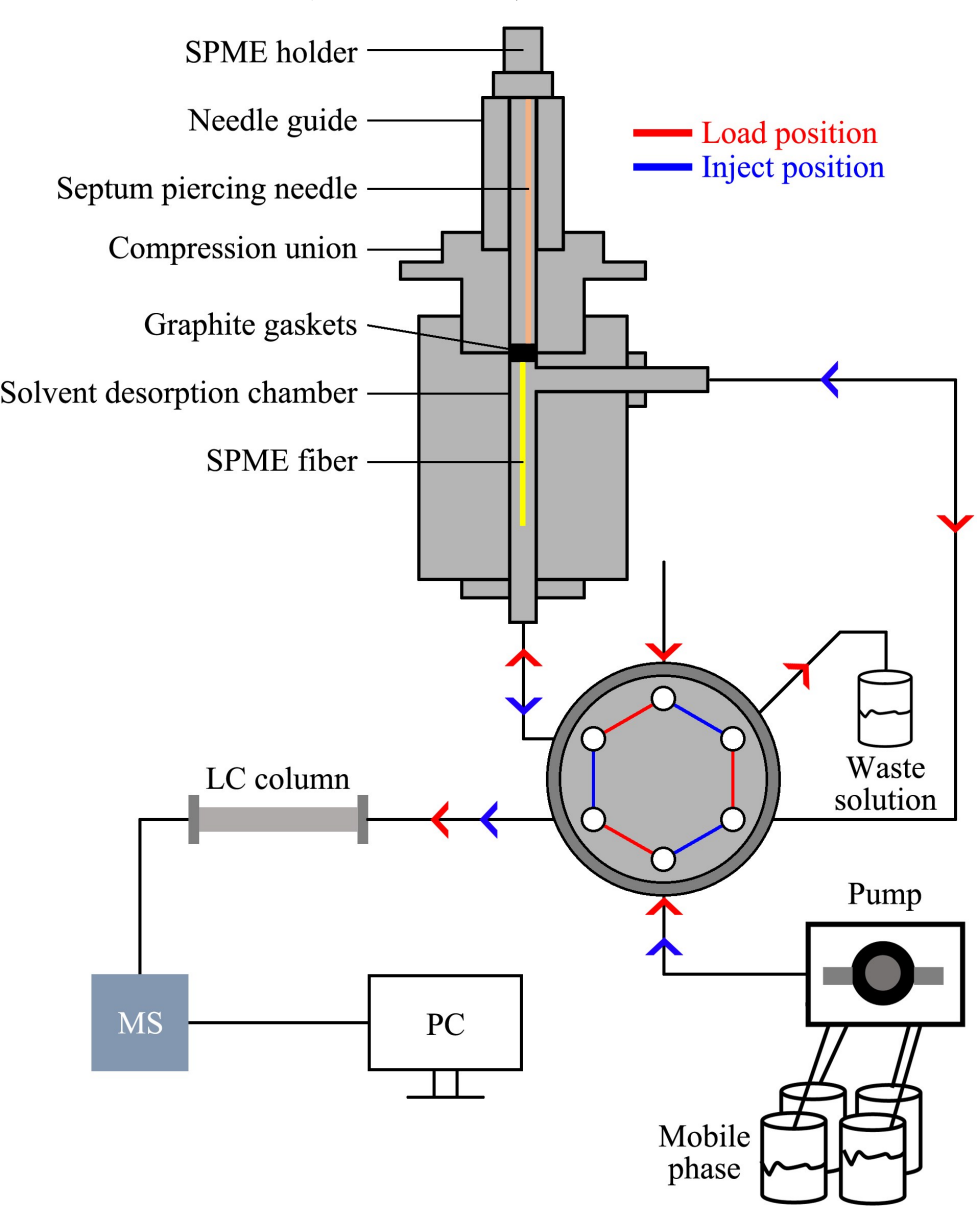
直接浸没-固相微萃取-液相色谱-质谱联用装置图^[46]^

目前,采用UAE和SPE作为HBCDs前处理方法的研究较多(如图3),占比为45%。根据样品基质的不同,选择适当的前处理方法尤为重要,例如固体样品多采用超声辅助萃取结合固相萃取多步处理,进行HBCDs的萃取和净化;对于液体样品中HBCDs的前处理方法应多考虑吸附剂的选择,在分离目标物与杂质的同时,对目标物进行富集。

**图3:**
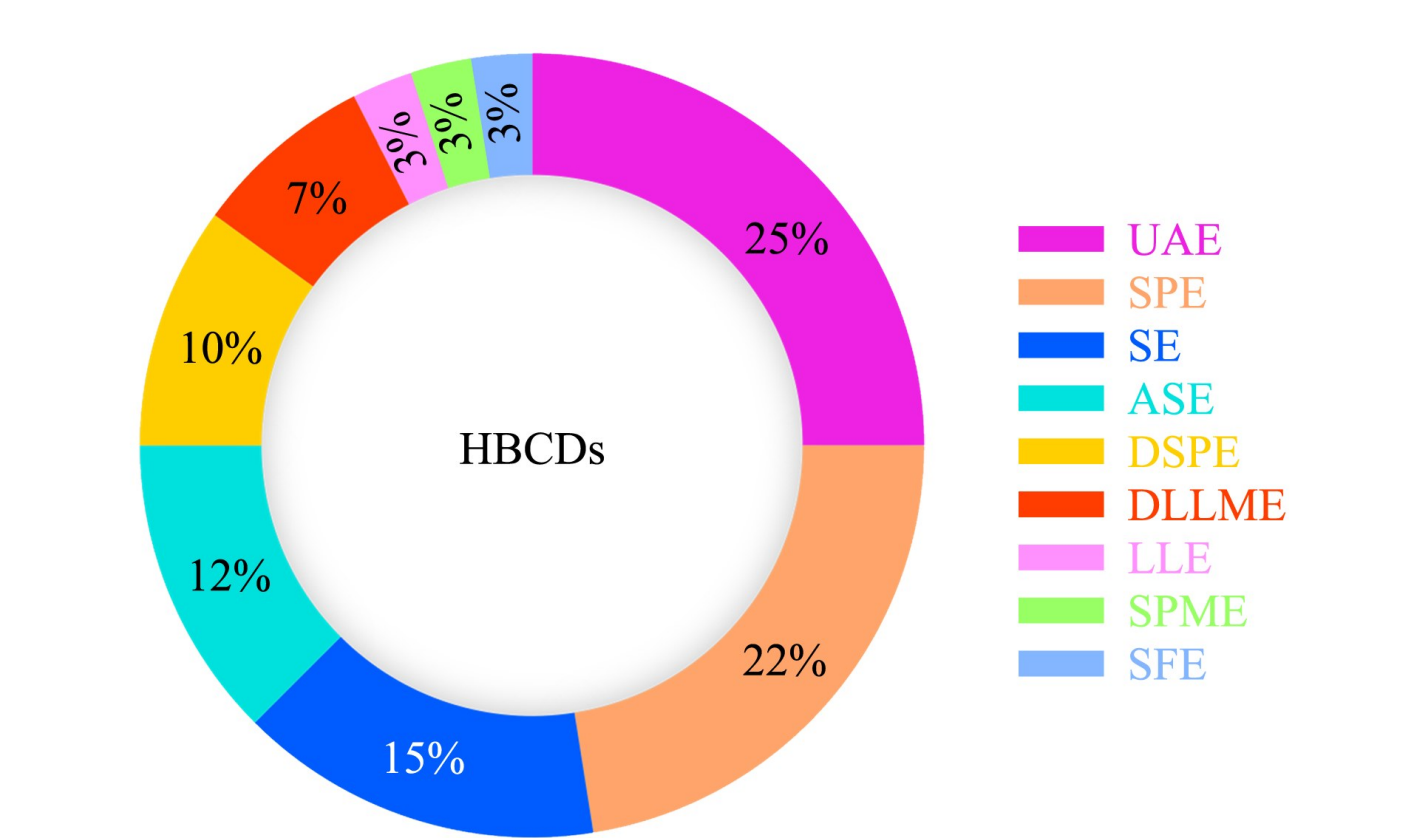
HBCDs样品前处理方法的统计(来源:Web of Science和中国知网数据库,2009~2022)

## 3 仪器检测方法

### 3.1 气相色谱法

HBCDs 3种异构体在160 ℃以上会相互转化,240 ℃以上会发生脱溴从而降解^[30]^,其热不稳定性为气相色谱法分析3种HBCDs异构体带来难度。部分研究采用该法对HBCDs总量(ΣHBCDs)进行分析。阮毅等^[53]^开发了碳骨架-气相色谱法对电子材料中ΣHBCDs进行测定。该法在进样口衬管中装入钯催化剂,在高温氢气体系内HBCDs被催化脱溴成已知目标物(C_12_直链烷烃),然后使用氢火焰离子化检测器测定,回收率为82.9%~106.0%, RSD小于5.6%。

### 3.2 气相色谱-质谱法

Chokwe等^[48]^将七氟丁酸(HFBA)作为HBCDs的衍生化试剂,结合气相色谱-质谱法测定沉积物中的HBCDs。经衍生化后,使用GC-MS可以测定含量为ng/g的HBCDs,提高了检测灵敏度。其中气相色谱柱初始温度为50 ℃,以7.5 ℃/min的速率升至120 ℃后,以15 ℃/min的速率升至275 ℃,再以25 ℃/min的速率升至300 ℃,停留2 min。将单四极杆质谱作为检测器,离子源为电子轰击离子源(EI),使用全扫描模式对目标物进行鉴定,最终该法的回收率为65%。

### 3.3 液相色谱法

对于HBCDs这类热不稳定物质,使用液相色谱-紫外检测更为合适。采用反相液相色谱检测同分异构体的关键在于流动相和色谱柱的选择。而样品基质成分复杂或前处理净化不完全,也会为液相色谱检测增加难度。Pursch等^[57]^为降低基质干扰,建立了二维液相色谱系统用于测定聚合物材料中HBCDs的含量(如图4所示)。该系统将一维柱(Zorbax SB-Phenyl, 150 mm×2.1 mm,1.8 μm)与二维柱(Zorbax SB C18, 50 mm×3.0 mm,1.8 μm)串联,样品进入到一维色谱柱时,对目标物出峰时间段的组分进行中心切割,将其存储在十二通阀中,随后再将阀内中心切割的组分注入第二维柱中,实现复杂基质中样品的定量分析。该法RSD为0.7%,可以通过一维和二维的结合减少基质与目标物的峰重叠,从而实现准确定性定量分析的目的^[57]^。当常规的一维液相色谱不能有效分离目标物时,二维液相色谱是可替代的解决方案。

**图4:**
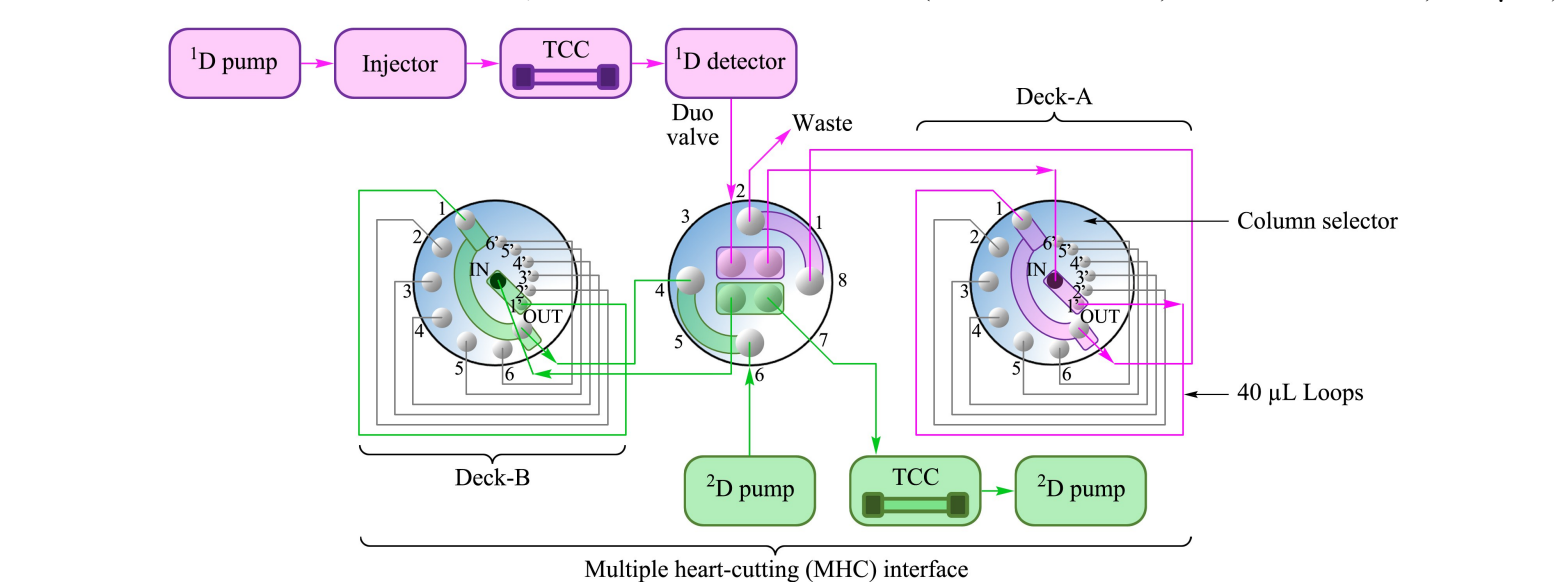
二维液相色谱系统图^[57]^

### 3.4 液相色谱-质谱法

#### 3.4.1 单四极杆

对于基质单一且HBCDs含量较高的样品来说,将单四极杆质谱作为检测器,足以对样品中的HBCDs进行测定。陈琼等^[33]^使用LC-MS测定电子电器产品中的HBCDs,对LC-MS检测条件进行优化时发现,在流动相里加入甲酸或者乙酸铵时可提高3种异构体的分离度和稳定性,但是会增加色谱柱的平衡以及冲洗时间,导致整个分析时间的延长。将甲醇-水作为流动相时,加大甲醇浓度可延后HBCDs的出峰时间以及增加其分离度,所以最终选定甲醇-水(88∶12, v/v)作为色谱的流动相。李岩等^[16]^对单四极杆质谱的选择离子监测(SIM)模式和选择反应监测(SRM)模式进行了考察。结果表明,两种模式的灵敏度相当,但SIM的稳定性(5%)高于SRM(8%),因此选择SIM模式对环境大气中的HBCDs进行测定。

#### 3.4.2 三重四极杆

液相色谱-串联质谱法是测定HBCDs最常用的仪器检测方法。使用三重四极杆质谱作为检测器,通过二级质谱轰击产生的碎片离子(*m/z* 79和*m/z* 81)作为定性和定量离子,可以提高方法的灵敏度,多项国标^[59,60]^和行标^[36,43,44,61]^均采用此方法。虽然工业生产中仅使用*α*、*β*、*γ*-HBCDs,但在沉积物、土壤或生物体内,已有其他不常见HBCDs(*δ*、*ε*、*ζ*、*θ*、*ι*、*κ*-HBCDs)的检出,这可能是因为在微生物或者生物作用下,3种主要的HBCDs发生转化。Baek等^[64]^针对10种HBCDs异构体建立了LC-MS/MS检测方法。他们测试了不同固定相(苯基-己基、氟-苯基和五氟苯基)的色谱柱对异构体的分离效果,结果表明,苯基-己基柱具有优异的分离效果,这可能与HBCDs和C6链具备的疏水作用有关。采用该方法在工业产品中检出了不常见的*δ*、*ε*、*η*、*θ*-HBCDs。

现阶段,针对不同的分析需求,可选择不同的仪器对HBCDs进行测定。对于测定ΣHBCDs的含量,可选择气相色谱或气相色谱-质谱。而液相色谱能够将多种HBCDs的异构体分离,对其进行准确的定性定量分析。选用质谱作为检测器时,三重四极杆质谱能够大大提高方法的灵敏度。

## 4 结论

目前,HBCDs在建筑行业中仍然大量使用,基于其高毒性和持久性,多国已做出严格限制,建立HBCDs的分析方法应对环境污染具有重要的意义。对于测定固体样品中HBCDs,需要将其从基质中提取后再进行净化,增加了样品前处理时间,降低了分析效率。而分析液体样品中HBCDs,通过SPE对其进行分离富集,可加大样品体积以提高富集倍率从而提高检测灵敏度。但HBCDs的前处理方法仍存在繁琐、耗时长、有机溶剂消耗量大等问题,基于新型吸附材料开发绿色环保、自动化、低成本,快速高效的新型样品前处理方法是未来这一领域的主要发展方向。气相色谱能够应用于测定HBCDs的总含量,而液相色谱-串联质谱技术有助于对多种HBCDs异构体进行定性定量分析。将新型样品前处理方法与适当的仪器检测方法结合,构建快速、高效、简单、准确的HBCDs分析方法对研究HBCDs在不同基质中的浓度分布、转化规律提供理论支撑,为评估HBCDs的环境健康风险和制定相关污染防控标准提供科学依据。
